# A greater incidence of nausea/vomiting reactions to Multihance^®^ is seen among those of African descent

**DOI:** 10.1186/1532-429X-17-S1-T6

**Published:** 2015-02-03

**Authors:** Kraig V Kissinger, Sophie Berg, Beth Goddu, Warren J Manning

**Affiliations:** 1 Cardiac MRI, Beth Israel Deaconess Medical Center, Boston, MA, USA

## Background

Gadolinium based contrast agents are frequently given to patients who undergo a Cardiac MRI examination to maximize contrast between normal and abnormal tissues in order to demonstrate pathologies such as scar or fibrosis in the heart and for MR angiography. Multihance^®^ (gadobenate dimeglumine, Bracco Diagnostics) is a common selection because of its properties of high T1 relaxivity. While contrast reactions are rare we have anecdotally observed that patients of certain racial backgrounds appear more likely to experience reactions of nausea or vomiting after Multihance^®^ infusion and sought to formally examine this possible relationship.

## Methods

We retrospectively identified 1773 consecutive CMR examinations over a 4 year period (2010-2014) utilizing Multihance^®^ from our CMR clinical database. These included 161 (9.1%: 58% male, 42% female) patients of self-described African descent. The remaining 1612 individuals were of other races, predominately Caucasian (68.6%: 63% male, 47% female) with a small minority of Asian (3.4%: 58% male, 32% female), Hispanic (4.9%: 64% male, 46% female) and other (0.5%: 25% male, 75% female) populations. An additional 240 individuals elected not to report ethnicity (13.5%: 75% male, 25% female) Patients were told that no preparation was required for their examination; therefore the time since their last meal was unknown. All individuals were monitored in the same way during their study including ECG, non-invasive blood pressure and respiratory monitoring. Verbal communication was maintained with all individuals before, during and after the administration of the contrast agent.

## Results

**Figure 1:**
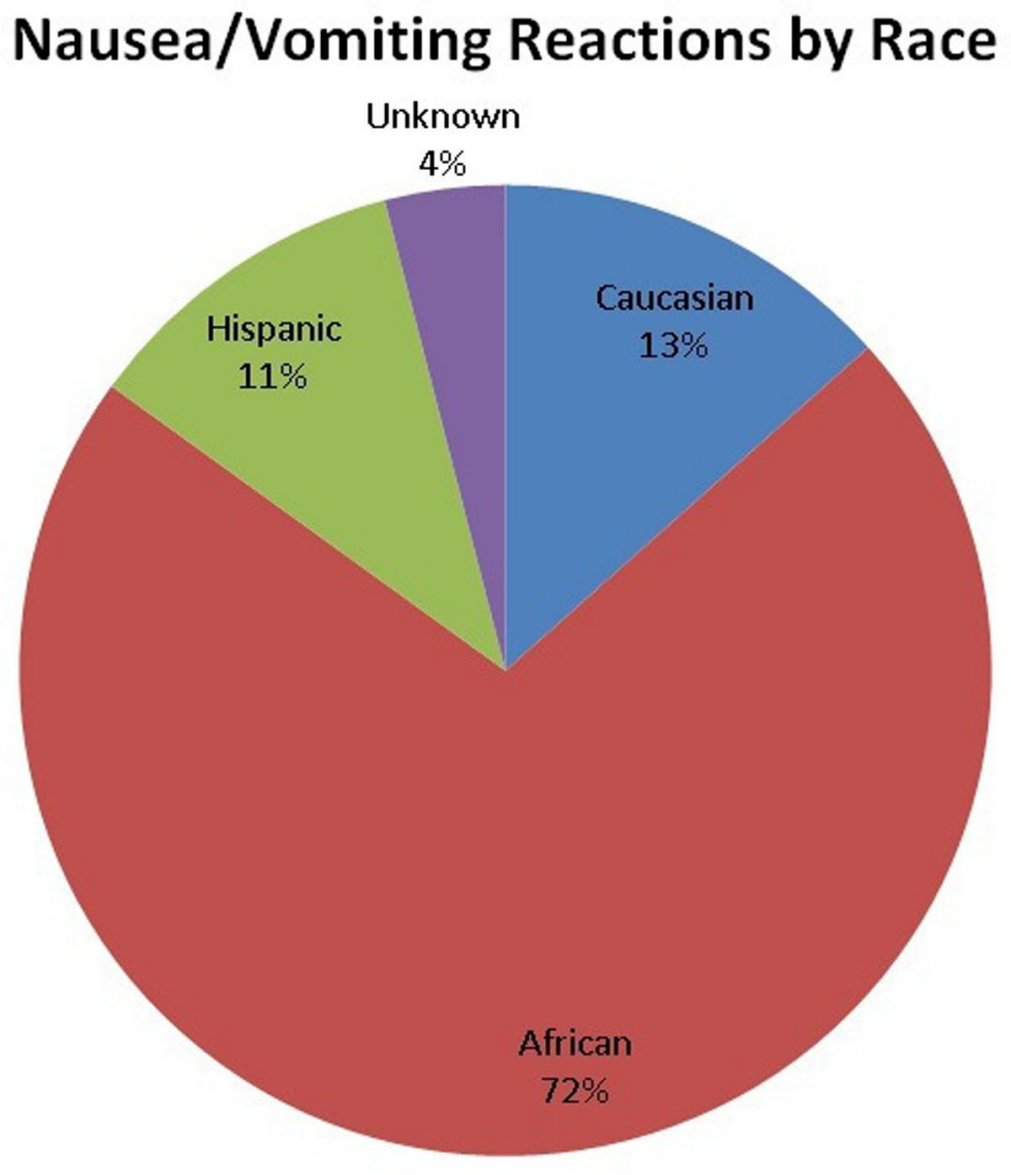


There were a total of 31 (1.75%: 77% male, 23% female) reactions involving nausea and vomiting immediately after Multihance^®^ infusion including 12 (7.5%: 83% male, 17% female) African descent, 17 (1.4%: 71% male, 29% female; p<0.0001 vs. non-African descent) Caucasian, 1 (1.5%: 100% male, 0% female) Hispanic and 1 (0.42%: 100% male, 0% female) of unknown race/ethnicity (Figure 1).

## Conclusions

In this consecutive, retrospective study of patients referred for clinical CMR study, we found a much higher incidence of nausea/vomiting reactions to the Multihance^®^ brand of gadolinium based contrast agent in individuals of African descent as compared to individuals who identify themselves as of non-African descent. Further prospective studies are needed to determine if this finding remains consistent and if ethnicity should be considered in the choice of CMR contrast agent.

## Funding

N/A.

